# 4445 Using Exit Interviews as One Component of the KL2 Program Impact Analysis Method

**DOI:** 10.1017/cts.2020.245

**Published:** 2020-07-29

**Authors:** Shaweta Singla, Oluwamuyiwa Winifred Adebayo, Karen Shields, Lorah Dorn, Diane Thiboutot

**Affiliations:** 1 Penn State Clinical and Translational Science Institute; 2 The Pennsylvania State University

## Abstract

OBJECTIVES/GOALS: The Penn State KL2 Career Development Program provides a comprehensive structured training and mentorship to junior faculty scientists (KL2 scholars). The goal of this study is to describe the perceptions of scholars after completion of the training and determine self-perceived impact of the program using exit interviews as a unique method. METHODS/STUDY POPULATION: Ten KL2 scholars (5 from each cohort of 2014 and 2017) participated in the evaluation. We used a descriptive qualitative design supplemented with quantitative data, to conduct an individual in-depth exit interview with each scholar to understand their perceptions on the impact of the KL2 program. Data were collected using a semi-structured interview guide developed by the program directors including scholars and a Likert scale survey. Thematic analysis of the data involved: reading and re-reading transcripts, identifying and categorizing keywords and phrases and developing overall themes that explained the processes within categories. In establishing rigor, two authors carefully coded, categorized and identified patterns and emerged themes which were also reviewed and confirmed by the other authors. RESULTS/ANTICIPATED RESULTS: Two sets of themes emerged. The main themes that described positive aspects of the KL2 program by scholars included: *Interdisciplinary Collaboration, Mentoring, and Protected Time for Independent Research*. Scholars also identified some contrary themes that included: *Limited Access to Expenditures, Changes in Individual Mentorship Needs and Areas for Improvement*. On a Likert scale (1- not at all, 10-extremely likely), scholars reported high positive influence of the KL2 program on their scope of research (8.7±0.52) and future career (8.5±0.70). They also found mentorship experience with primary mentor (9.6±0.22) and team (8.5±0.54) as well as peer collaboration (8.5±0.67) opportunities highly beneficial to their career and professional development. DISCUSSION/SIGNIFICANCE OF IMPACT: The qualitative study strengthens the reliability of data and scholar recommendations collected via other evaluation measures. Findings broaden understanding of the processes through which program outcomes are achieved effectively and where modifications are needed. An updated program for cohort 3 was guided by cohort 1 and 2 interview responses.

